# Developmental thyroid disruption causes long-term impacts on immune cell function and transcriptional responses to pathogen in a small fish model

**DOI:** 10.1038/s41598-021-93929-8

**Published:** 2021-07-14

**Authors:** Leah M. Thornton Hampton, Miranda G. Finch, Christopher J. Martyniuk, Barney J. Venables, Marlo K. Sellin Jeffries

**Affiliations:** 1 Department of Biology, Texas Christian University, 2800 S. University Dr., Fort Worth, TX 76129 USA; 2 Department of Biological Sciences, University of North Texas, Denton, TX USA; 3 Department of Toxicology, Southern California Coastal Water Research Project, Costa Mesa, CA USA; 4 Center for Environmental and Human Toxicology, Department of Physiological Sciences, College of Veterinary Medicine, UF Genetics Institute, Interdisciplinary Program in Biomedical Sciences Neuroscience, University of Florida, Gainesville, FL USA

**Keywords:** Gene regulation in immune cells, Innate immune cells, Innate immunity, Immunological techniques, Gene expression profiling, Developmental biology, Endocrinology

## Abstract

Current evidence suggests thyroid hormones (THs) impact development of the immune system, but few studies have explored the connection between the thyroid and immune systems, especially in fish. This is important as some environmental contaminants disrupt TH homeostasis and may thus have negative impacts on the immune system. To determine the long-term consequences of early life stage (ELS) hypothyroidism on immune function, fathead minnows were exposed to the model thyroid hormone suppressant propylthiouracil (PTU) from < 1 to 30 days post hatch. Fish were transferred to clean water and raised to adulthood (5–7 months post hatch) at which time, several aspects of immune function were evaluated. Ex vivo assessment of immune cell function revealed significant decreases (1.2-fold) in the phagocytic cell activity of PTU-treated fish relative to the controls. Fish were also injected with *Yersinia ruckeri* to evaluate their in vivo immune responses across a suite of endpoints (i.e., transcriptomic analysis, leukocyte counts, spleen index, hematocrit, bacterial load and pathogen resistance). The transcriptomic response to infection was significantly different between control and PTU-treated fish, though no differences in bacterial load or pathogen resistance were noted. Overall, these results suggest that early life stage TH suppression causes long-term impacts on immune function at the molecular and cellular levels suggesting a key role for TH signaling in normal immune system development. This study lays the foundation for further exploration into thyroid-immune crosstalk in fish. This is noteworthy as disruption of the thyroid system during development, which can occur in response to chemicals present in the environment, may have lasting effects on immune function in adulthood.

## Introduction

The connection between the thyroid and immune systems is well recognized, but only partly understood^1,2^. Thyroid hormones (THs) and thyroid stimulating hormone (TSH) influence a wide variety of immunological processes such as antibody production, lymphocyte proliferation, phagocytosis, and respiratory burst in mammals^2^. Among fish, thyroid follicles have been documented in the head kidney, a major immune organ in fish, suggesting the potential for localized crosstalk between TH-producing cells and immune cells^3^. Further, TH receptors α (*trα*) and β (*trβ*) are expressed in primary immune organs and cells (i.e., head kidney, spleen, isolated leukocytes) of rainbow trout (*Oncorhynchus mykiss*), and their expression can be significantly altered in the spleen and in isolated leukocytes following treatment with exogenous TH or propylthiouracil (PTU, a model thyroid suppressant that inhibits thyroid peroxidase activity in fish)^4^. These treatments also induced alterations in leukocyte subpopulations^3^ and the expression of genes/pathways related to interferons, complement, macrophage function, interleukins, viral defense, etc.^5^, suggesting that thyroid-immune crosstalk exists not only in mammalian systems, but in fish as well.

Thyroid hormones may also play a role in immune system development as studies in mammals have shown that thyroid suppression during early development induces alterations in the spleen and thymus, including decreases in tissue weight and shifts in lymphocyte subpopulations^6–8^. Similar observations have also been made in zebrafish (*Danio rerio*) exposed to methimazole (MMI, a model thyroid suppressant) from 5 to 28 days post fertilization (dpf), where fish exposed to MMI experienced decreases in thymus volume as well as the downregulation of transcripts involved in lymphocyte development and maturation^9^. Moreover, recent studies in mice have provided evidence that hypothyroid conditions during development may have permanent, long-term consequences on immune function and the immune response. Specifically, adult female mice gestated under hypothyroid conditions were found to have significantly increased survival following *Streptococcus pneumoniae* infection^10^ or increased severity of experimental autoimmune encephalomyelitis^11,12^.

Despite evidence demonstrating both the sensitivity of early life stage (ELS) fish to contaminant-induced thyroid disruption^13^ and the crosstalk between the thyroid and immune systems in fish^4,5,9^, to our knowledge, there are no studies that explore the potential long-term consequences of ELS thyroid disruption on immune function in fish. Therefore, it is important to consider the developing immune system as a potential indirect target of thyroid disrupting chemicals.

**Figure 1:**
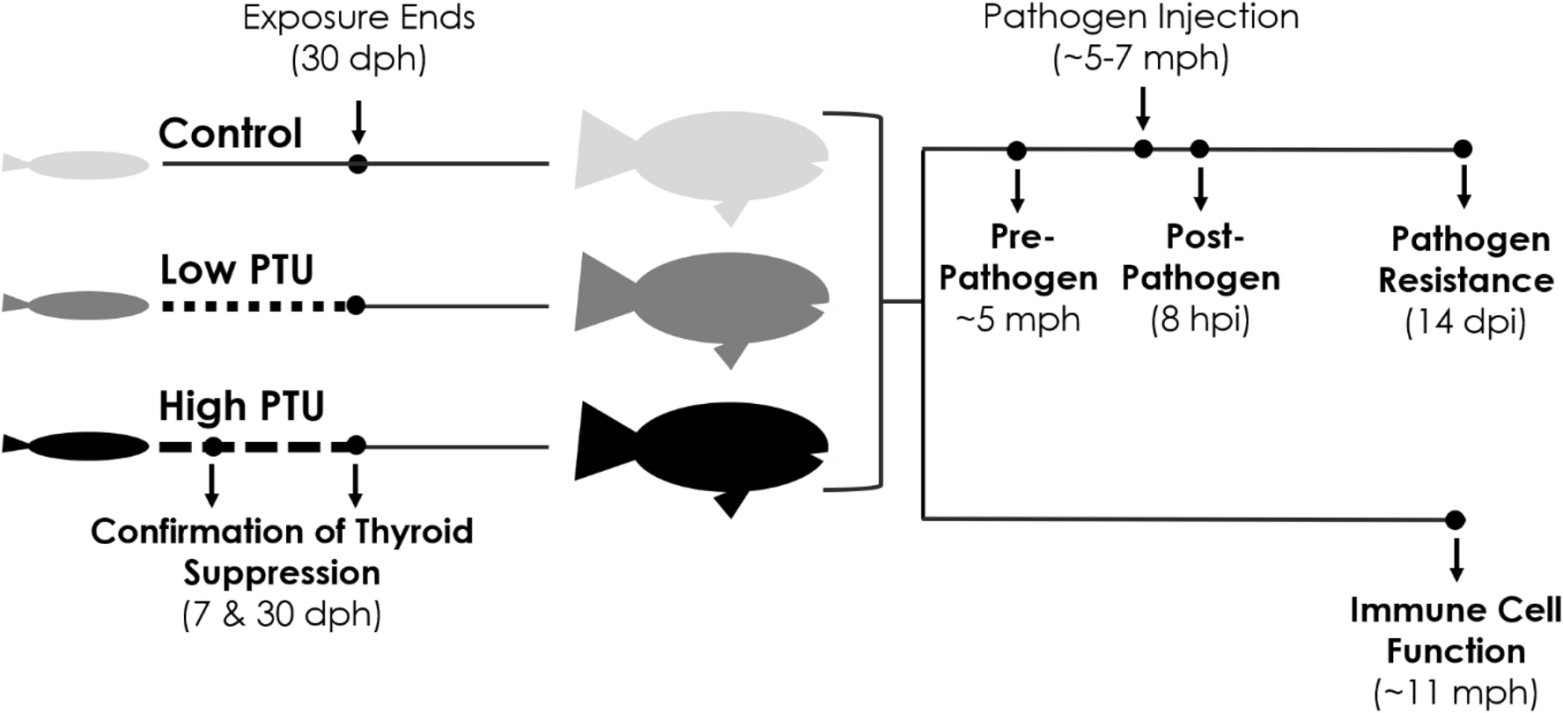
General experimental design of the current study. Fathead minnow larvae (< 1 day post hatch, dph) were exposed to either a low (25 mg/L) or high (70 mg/L) concentration of propylthiouracil (PTU), a model thyroid hormone suppressant, through 30 dph. Thyroid hormone (TH) suppression was confirmed via immunofluorescent labeling of thyroxine and TH-sensitive gene expression analysis at 7 and 30 dph, respectively. In addition, growth (i.e., mass, length) was assessed at both time points. Upon reaching adulthood at ~ 11 months post hatch (mph), ex vivo cellular immune function, specifically respiratory burst and phagocytic cell activity, was assessed. The in vivo immune response was determined via the assessment of a suite of immune-related endpoints across multiple levels of biological organization (i.e., transcriptomic analysis, leukocyte counts, spleen index, hematocrit, bacterial load, and pathogen resistance) following injection with *Yersinia ruckeri*.

The goal of this study was to determine the long-term consequences of ELS hypothyroidism on immune function and the immune response in a widely-used ecotoxicological model species, the fathead minnow (*Pimephales promelas*). To achieve this goal, fish were exposed to PTU from < 1 to 30 days post hatch (dph) and then raised to adulthood in clean water. Thyroid hormone suppression was confirmed via the immunofluorescent labeling of thyroxine (T4) at 7 dph and the analysis of TH-sensitive transcripts in liver tissue at 30 dph. Upon reaching adulthood, alterations in cellular immune function were determined via the assessment of phagocytic cell activity and respiratory burst ex vivo. To address potential differences in adult immune responses, fish were injected with *Yersinia ruckeri* and a suite of immune-related endpoints across biological levels of organization were evaluated (i.e., transcriptomics analysis, leukocyte counts, spleen index, hematocrit, bacterial load and pathogen resistance) (Fig. 1). These endpoints were selected given their sensitivity to *Y. ruckeri* infection and their role in the innate immune response in fish^14–16^.

## Results

### Confirmation of thyroid suppression

**Figure 2:**
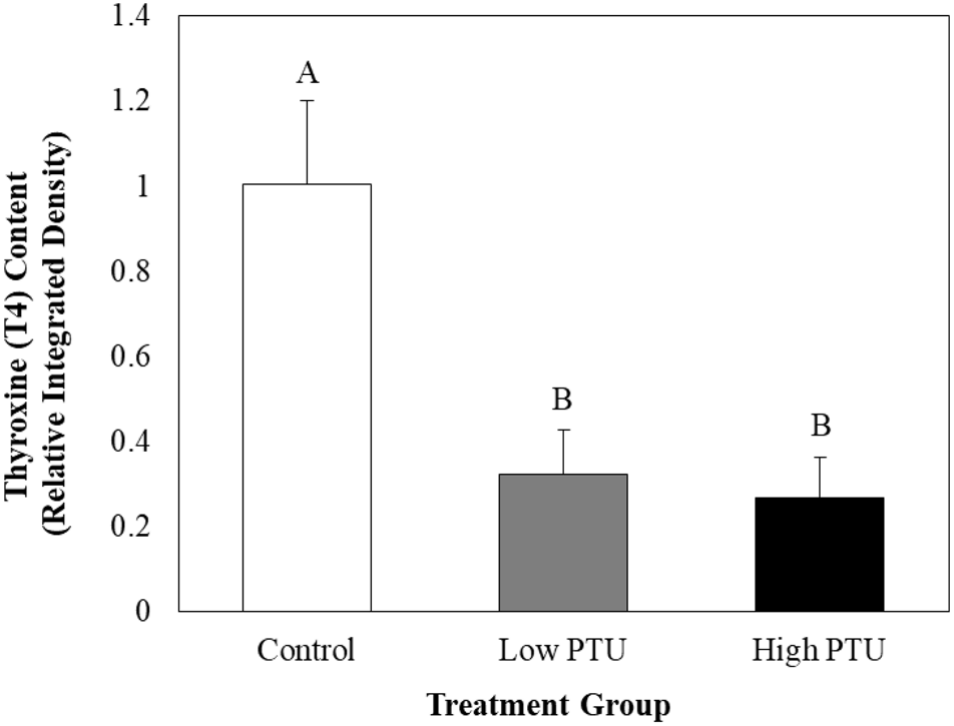
Thyroxine (T4) content in the follicular cells of control and propylthiouracil (PTU)-exposed larvae at seven days post hatch as measured by integrated density (n = 10). Values represent mean integrated density in PTU-exposed larvae relative to control larvae. Error bars represent standard error.

**Figure 3:**
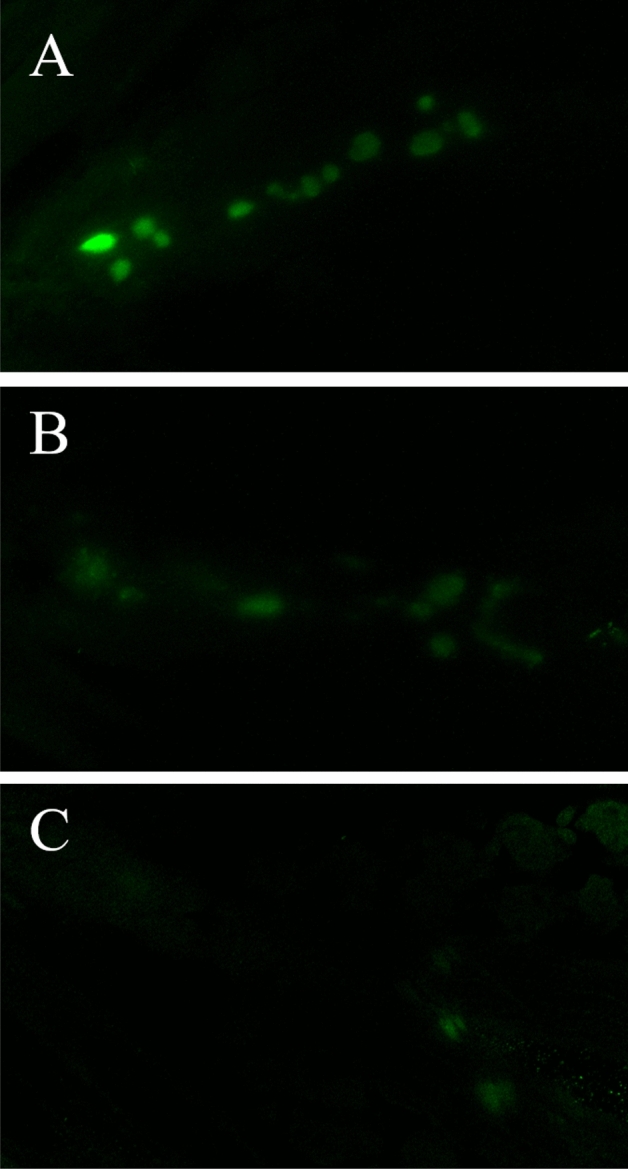
Representative images of immunofluorescent labeling of thyroxine (T4) in control (**A**), low concentration (**B**) and high concentration propylthiouracil-exposed larvae (**C**).

Significant reductions in T4 content, as measured by relative integrated density (ID), were detected between groups at 7 dph (Fig. 2, ANOVA, p-value < 0.01, df = 2, F statistic = 8.67). Fish in both the low- and high-PTU groups experienced significant decreases in ID (68 and 73%, respectively) relative to the control. Representative images of fluorescently-labeled thyroid follicles are presented in Fig. 3.

**Figure 4:**
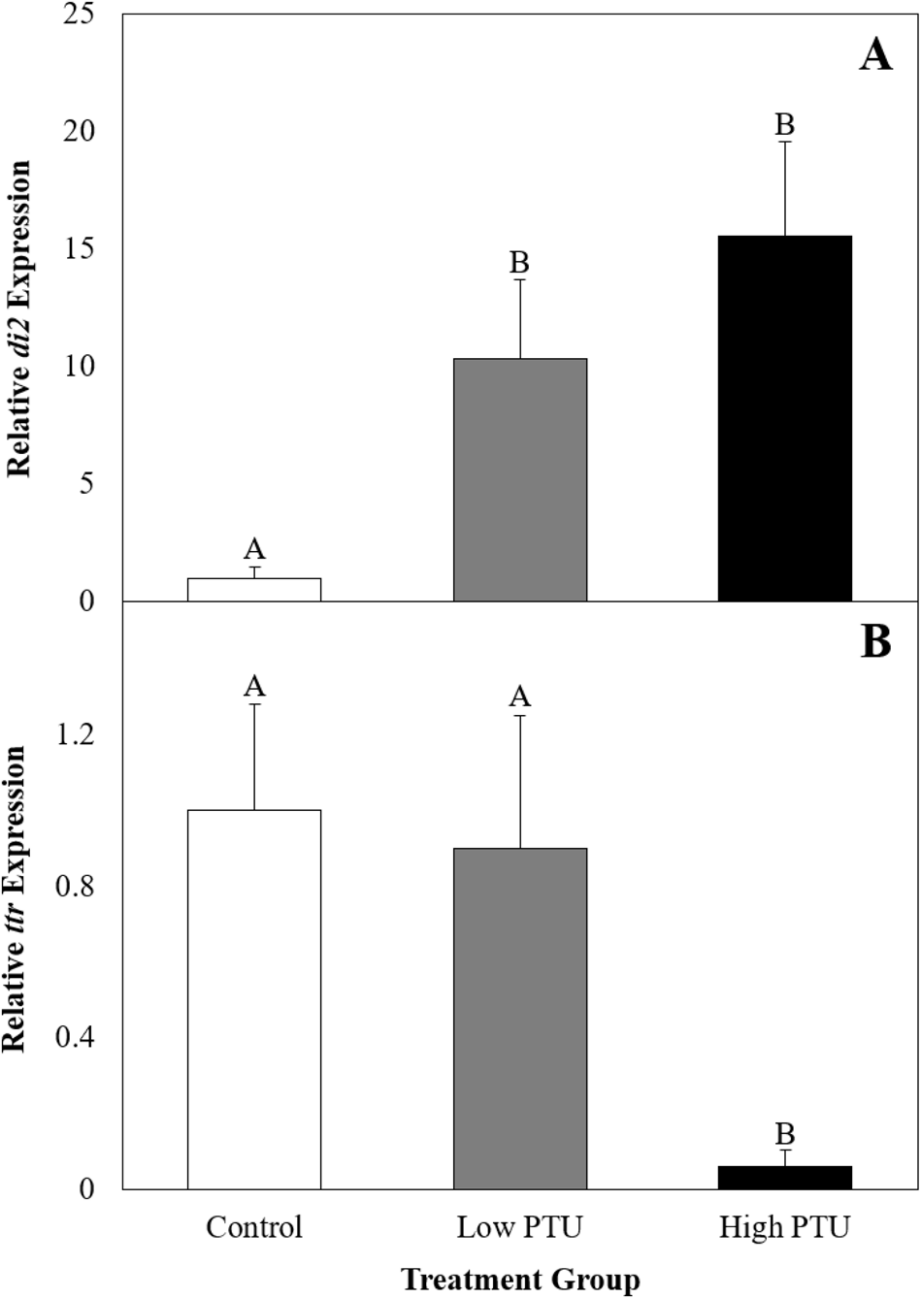
Hepatic expression of *deiodinase 2* (*di2*, A) and *transthyretin* (*ttr*, B) in control and propylthiouracil (PTU)-exposed larvae at 30 days post hatch (n = 8). Values represent mean fold change in PTU-exposed larvae relative to control larvae. Error bars represent standard error. Different letters indicate statistically significant differences between treatment groups.

At 30 dph, significant differences in the relative hepatic expression of both *di2* and *ttr* were detected between groups (Fig. 4, Kruskal–Wallis, both p-values < 0.01, df = 2). Relative to the controls, expression of *di2* was significantly elevated by 10 and 16 fold in the low and high PTU groups, respectively (Fig. 4A); whereas *ttr* expression was significantly downregulated by 16 fold in the high-PTU group relative to the control group (Fig. 4B).

No significant differences were detected in growth (i.e., mass and length) at 7 dph [Table S3, ANOVA, both p-values ≥ 0.05, df = 2, F statistics = 3.26 (mass), 1.16 (length)]. However, significant reductions in mass and length were detected at 30 dph [Table S1, ANOVA, both p-values < 0.01, df = 2, F statistic = 18.04 (mass), 32.62 (length)]. Fish in the high-PTU group had significantly reduced mass (2.6 fold) and length (1.4 fold) compared to those in the control and low-PTU groups, whereas the low-PTU fish had significantly reduced length (1.1 fold) relative to only the controls.

### Immune cell function

**Figure 5:**
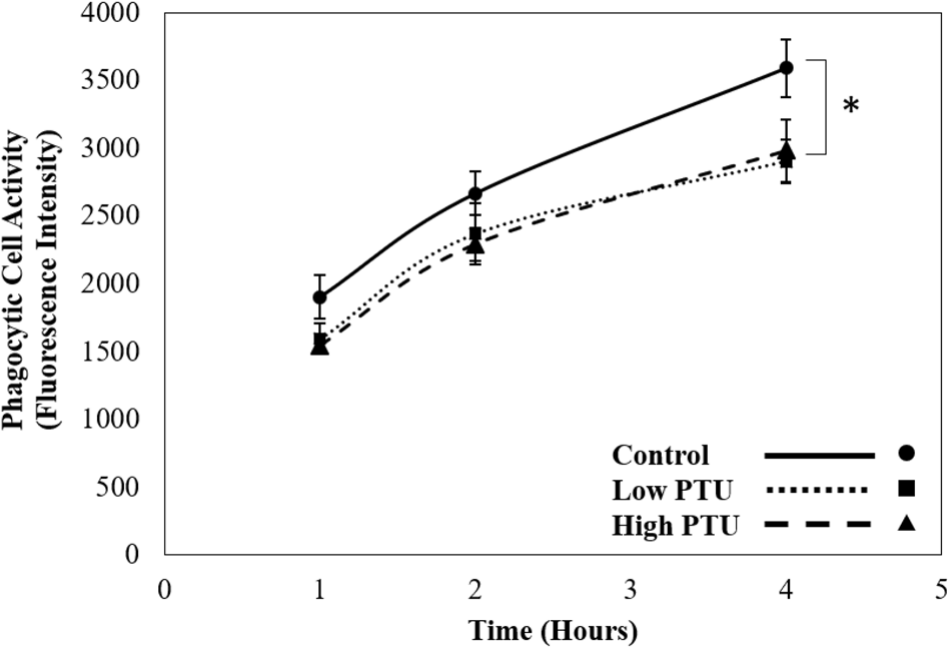
Mean phagocytic cell activity in renal cells of control and propylthiouracil (PTU)-exposed fish (n = 5/group). Error bars represent standard error. Asterisk indicates statistically significant difference between control and PTU treatment groups.

At 11 months post hatch, a subset of fish were utilized for the assessment of immune cell function. There were no significant differences in the masses nor lengths of control, low-PTU or high-PTU fish at this time point (Table S2). No significant differences between groups were detected for respiratory burst in either unstimulated (Table S3, Kruskal–Wallis, p-value = 0.11, df = 2) or stimulated cells (Table S3, ANOVA, p-value = 0.65, df = 2, F statistic = 0.45). However, analysis of phagocytic cell activity revealed that there was both a significant effect of time and treatment group (Fig. 5, two-way ANOVA, both p-values ≤ 0.01, df = 8, F statistic = 16.39). Specifically, phagocytic cell activity increased with increasing incubation time, and both PTU groups had decreased (1.2 fold) phagocytic cell activity relative to the control group.

### Renal transcriptomic response to pathogen infection

When comparing the renal transcriptome across treatment groups, 190 differentially-expressed transcripts, corresponding to 87 unique genes, were identified (p-value < 0.05, FDR-corrected and log_2_FC < − 1 or > 1). Cluster analysis of differentially-expressed genes (DEGs) revealed three distinct clades corresponding to each treatment group (Fig. S1).

Pathway analysis revealed that 207 and 234 gene networks were significantly altered in the low- and high-PTU treatment groups, respectively, when compared to the control group. Among these were networks directly related to immune processes and cell types, as detailed in Table 1. One immune-related network, monocyte activity, was found to be upregulated among low-PTU fish. Among the high-PTU fish, five immune networks were upregulated (iron absorption, macrophage response, neutrophil homeostasis, opsonization, and transepithelial migration), while three were downregulated (antigen binding, cellular immune response and colony formation) (Table 1).

**Table 1:** Significantly enriched immune-related subnetworks following *Yersinia ruckeri* infection in propylthiouracil (PTU)-exposed fish relative to controls.

Gene network	Median fold change	Number of genes measured	Network coverage (%)
**Low PTU**			
Monocyte activity	+ 1.39	17	45
**High PTU**			
Antigen binding	− 1.14	50	40
Cellular Immune Response	− 1.05	285	34
Colony formation	− 1.38	1183	36
Iron absorption	+ 1.42	30	45
Macrophage response	+ 1.38	46	34
Neutrophil homeostasis	+ 1.67	12	43
Opsonization	+ 1.38	27	24
Transepithelial migration	+ 1.35	31	37

### Validation of transcriptomic results

Regression analysis between relative gene expression measured via qPCR analysis and counts per million (CPM) determined via RNA sequencing revealed significant correlations for four of the five genes selected for validation—*csfr1*, *helz2*, *rn223* and *ubi1p* (Fig. S2, regression analysis, all R^2^ ≥ 0.40, all p-values ≤ 0.03). The relative expression and CPM of *smox* were not significantly correlated (Fig. S2, regression analysis, R^2^ = 0.10, p-value = 0.32).

### Other immune endpoints and pathogen resistance

**Figure 6:**
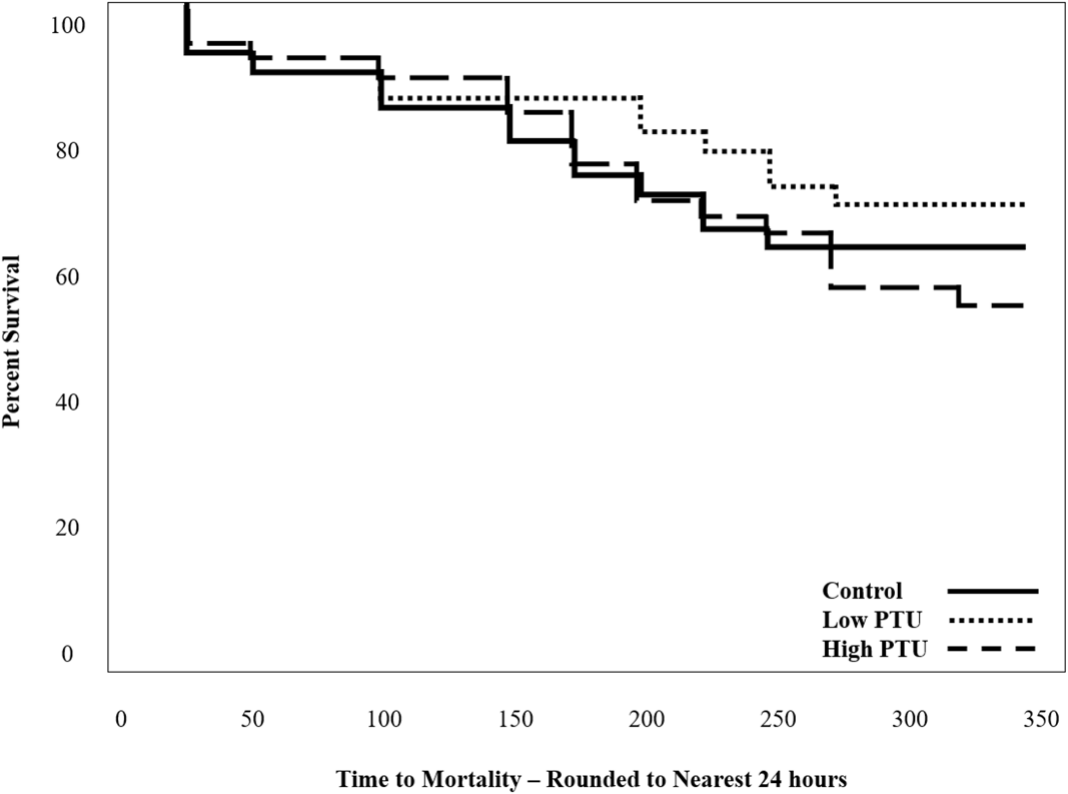
Survival curves of control and propylthiouracil (PTU)-exposed fish following pathogen injection (n = 3 trials, each consisting of 12 fish/group).

Immediately following pathogen injection, the size of each fish was evaluated and no significant differences in mass nor length were detected between groups [Table S4, ANOVA, p-values = 0.45 (mass), 0.24 (length), df = 2, F statistic = 0.82 (mass), 1.50 (length)]. No significant differences in pathogen resistance, as measured by percent survival following pathogen injection, were noted between groups (Fig. 6, survival time analysis, p-value = 0.18, df = 2). In addition, no differences were detected between groups for any of the innate immune function endpoints measured at 8 h post pathogen injection (i.e., spleen index, leukocyte counts, hematocrit, and bacterial load) [Table 2, ANOVA, all p-values ≥ 0.32, df = 2, F statistics = 0.96 (spleen index), 1.67, 0.25 (pre and post pathogen leukocyte counts), 1.18 (hematocrit), 0.59 (bacterial load)].

**Table 2:** Immune response parameters (mean ± standard error) measured in control and propylthiouracil (PTU)-exposed fish following pathogen injection (n = 9–12/group).

Endpoint	Control	Low PTU	High PTU
Spleen Index	0.21 ± 0.03	0.17 ± 0.01	0.18 ± 0.01
Leukocytes (%)	3.48 ± 0.43	3.38 ± 0.60	2.83 ± 0.94
Hematocrit (% erythrocytes)	45.92 ± 5.26	53.13 ± 3.15	53.69 ± 3.30
Bacterial Load (*yr16s* expression)	1.00 ± 0.22	1.42 ± 0.42	1.65 ± 1.00

*yr16S Yersinia ruckeri* 16S ribosomal subunit.

## Discussion

The main goal of this study was to determine the long-term consequences of chemically-induced ELS hypothyroidism on immune function using the fathead minnow as a model. Though no alterations in overall pathogen resistance were observed, transcriptomic analysis revealed distinct patterns of immune-related gene expression in each of the PTU treatment groups relative to the controls following infection with *Y. ruckeri*. Fish subjected to ELS hypothyroidism also had impaired immune cell function as indicated by significant reductions in renal phagocytic cell activity. Together, these results suggest that thyroid suppression during early development imparts long-term alterations on the immune system at the molecular and cellular levels.

### Propylthiouracil exposure successfully suppressed thyroid function in larval fish

In the current study, TH suppression was confirmed via the assessment of T4 content, thyroid-related gene expression, and growth. Reductions in T4 content were expected as PTU directly inhibits TH synthesis^17^, and similar exposures have been shown to cause reductions in follicular T4 content in zebrafish (*Danio rerio*) using comparable analytical methods^18^. Here, the induction of *di2* and downregulation of *ttr*, which have been previously observed in larval fathead minnows exposed to PTU^19^, may be interpreted as compensatory mechanisms to promote the synthesis of biologically-active TH and increased bioavailability of THs, respectively. Finally, decreases in mass and length, like those observed among 30 day old PTU-exposed fish in the current study, are well-documented in fish following goitrogen exposure^9,20^. It should be noted that changes in growth did not persist into adulthood, as there were no differences between the masses nor lengths of adult fish utilized to evaluate immune function, regardless of their exposure history. This suggests that once transferred to clean water, the somatic development of PTU-exposed fish is restored. Thus, any differences in immune parameters measured in adult fish are not a result of differences in somatic development or size.

### Developmental thyroid suppression alters the transcriptomic immune response to Yersinia ruckeri

Immune-related gene networks were significantly enriched in both PTU treatment groups following infection with *Y. ruckeri*, indicating that ELS TH suppression altered the transcriptomic immune response. Interestingly, there was no overlap in the enriched networks between the low- and high-PTU groups when compared to the control group, suggesting that the observed alterations in gene expression are concentration specific. Broadly, significantly enriched networks were associated with cell migration, iron absorption, complement, and pathogen recognition. Most enriched gene networks were related to immune cell function and included a combination of both upregulated and downregulated networks, predominately in the high-PTU group. The downregulation of networks associated with cellular immune response and colony formation and the upregulation of networks related to monocyte activity, macrophage response, neutrophil homeostasis, and transepithelial migration are indicative of potential changes in cellular immune function. A recent study in ELS zebrafish also documented transcriptional changes in immune cell-related networks following exposure to PTU (e.g., activation of leukocytes, cell movement of neutrophils, development of helper T cells), though gene expression was analyzed immediately following exposure at 96 h post hatch^21^. In mammals, changes in immune cell function have been previously observed following developmental hypothyroidism. For instance, the migration of innate immune cells^10^ and differentiation of T cells^12^ have been shown to be altered following gestational TH suppression in mice. Alternatively, gene expression changes may be driven by differences in renal immune cell populations. Previous studies in mammals have noted alterations in the weight and cellularity of lymphoid tissues as well as shifts in immune cell subpopulations following ELS thyroid hormone suppression^6–8^. For example, pigs with congenital hypothyroidism had significantly reduced numbers of lymphocytes in peripheral blood^8^ and neonatal PTU exposure reduced leukocyte population sizes in the spleen and thymus of rats^6^. In the current study, it is unclear if the observed transcriptomic alterations are a result of altered immune cell function or changes in immune cell populations. Differentiation of these possibilities would require a characterization of renal tissue cell types or isolation of specific cell types prior to transcriptomic analysis.

### Developmental thyroid suppression causes decreases in phagocytic cell activity

Fish from both of the PTU treatment groups experienced significant decreases in phagocytic cell activity relative to those from the control group, indicating that phagocytosis had been permanently impacted by ELS TH suppression. As described previously, studies in mammals have noted changes in immune cell populations following ELS hypothyroidism^6–8^. Thus, it is possible that the noted reductions in phagocytic cell activity result from a decrease in the number of phagocytic cells in the renal tissue of PTU-exposed fish. However, this hypothesis remains to be tested as renal cell populations were not characterized in the current study. Alternatively, phagocytic cells from PTU-treated fish may be functionally impaired relative to those from the control group as differences in immune cell activities have been previously observed following ELS hypothyroidism^10,12^. Though the mechanisms are unclear, this is the first study to show reductions in phagocytic cell activity following ELS thyroid suppression in fish.

The previously described, transcriptomic changes related to immune cell function may provide evidence for altered phagocytic cell activity in PTU-exposed fish. Specifically, gene networks associated with phagocytic cell types were significantly enriched in each of the PTU groups following infection with *Y. ruckeri*. In the low PTU group, monocyte activity was upregulated and in the high-PTU group, macrophage response and neutrophil homeostasis were upregulated. Monocytes, macrophages and neutrophils are considered “professional” phagocytes in mammals, as well as in fish^22,23^ making it possible that these gene networks are upregulated in the PTU-exposed fish as a compensatory mechanism to promote phagocytic cell activity and/or recruitment of phagocytic cells. For instance, the gene networks for monocyte activity and macrophage response both include the upregulated genes *c–c motif chemokine ligand 2* (11.9 fold) and *colony stimulating factor 1* (2.1 fold), each of which promotes the differentiation and recruitment of phagocytes^24,25^. Though these changes in gene expression are consistent with the results of the phagocytic cell assay, additional studies are required to fully explain the relationship between the observed molecular and cellular alterations of the current study.

### Developmental thyroid suppression does not alter immune responses beyond the molecular and cellular level

Though transcriptional responses to pathogens and phagocytic cell activity were altered as a result of ELS TH suppression, none of the other immune-related endpoints measured (i.e., respiratory burst, leukocyte counts, spleen index, hematocrit, bacterial load and pathogen resistance) were different from controls. This provides some evidence that the impacts of developmental hypothyroidism do not manifest beyond the molecular and cellular levels. The lack of alteration in higher-level endpoints measured here could be explained by the inherent redundancy and diversity of immune system responses, as a wide variety of host defense strategies are used to recognize and destroy invading pathogens during infection. Therefore, it is possible that the reductions in phagocytic cell activity were not so severe that other immune defense mechanisms could not adequately compensate. It is also possible that ELS hypothyroidism does lead to higher-order alterations in immune function, as has been seen in mammals^11,12^, but that the use of a single species of bacteria (*Y. ruckeri*) at a single dose precluded the detection of such changes. It is possible that infection with a virus, parasite or other bacterium would yield different results.

### Conclusions

The results of the current study demonstrate that female fathead minnows exposed to PTU from < 1 to 30 dph experience alterations in the transcriptomic response to *Y. ruckeri* and decreases in ex vivo phagocytic cell activity. However, no other alterations in immune function were observed for the selected immune endpoints, suggesting that the effects of developmental hypothyroidism do not manifest beyond the cellular level. However, fish were challenged with only a single species and dose of bacteria, and the results of this study may have differed if fish were infected with a different pathogen. It is also important to consider that this study focused on the activated immune system as all immune-related endpoints were evaluated following exposure to an immune stimulus (i.e., *Y. ruckeri*, *E. coli*, PMA), and it is currently unknown if the observed effects are a result of differences in baseline immunity or the immune response. Nevertheless, this is the first study to explore the long-term consequences of ELS hypothyroidism on immune function in fish. Future studies should seek to further describe crosstalk between the endocrine and immune systems throughout all stages of development. Immune assessments following exposure to environmentally-relevant thyroid disrupting chemicals are also recommended. Though more information is required to further describe thyroid-immune crosstalk and more fully understand the effects of thyroid-disrupting compounds on immune function in fish.

## Methods

### Animal husbandry and exposure regime

All experimental procedures involving fathead minnows were carried out in accordance with a protocol approved by the Texas Christian University (TCU) Institutional Animal Care and Use Committee (protocol # 17/13) and study information has been reported in accordance with ARRIVE guidelines. At < 1 dph, 892 larvae from the TCU fathead minnow colony were randomly sorted into groups (i.e., control, low PTU or high PTU). Exposure solutions were prepared daily by dissolving PTU in approximately 1 L dechlorinated municipal water in a 2L volumetric flask and placing the solution on a stir plate in an incubator (27 °C) overnight to ensure complete dissolution. Each morning, solutions were diluted to a final volume of 2L in the same volumetric flask. The solution was then transferred to a 10L carboy and diluted to achieve final concentrations of 25 and 70 mg PTU/L. From 0 to 14 dph, larvae were housed in 1L beakers at a density of 59–60 larvae/L until 15–30 dph when density was reduced to 29–30 larvae/L. All beakers were housed in a 26 °C water bath under a photoperiod of 16 h light:8 h dark. Uneaten food and wastes were removed daily during 80% water changes. Larvae were fed live *Artemia nauplii* twice daily in excess (Table S5). Renewal water (dechlorinated municipal tap water) had the following water quality characteristics (mean ± standard deviation): pH 7.8 ± 0.2; alkalinity (expressed as ppm CaCO_3_), 116.3 ± 8.2; hardness (expressed as ppm CaCO_3_), 108.0 ± 8.2; and conductivity 334.0 ± 35.8 μS/cm.

At 30 dph, larvae were moved to 30L aquaria filled with clean, dechlorinated tap water at a density of ~ 6.4 larvae/L. Over the following month, larvae were gradually transitioned to a diet of commercially-available flake food (Tetramin, Blacksburg, VA) and density was gradually reduced to ~ 1.3 larvae/L. Uneaten food and wastes were removed during daily ~ 30% water changes. Renewal water had the following water quality for the remainder of the experiments (mean ± standard deviation): pH 8.2 ± 0.2; alkalinity (expressed as ppm CaCO_3_), 90.5 ± 28.4; hardness (expressed as ppm CaCO_3_), 130.9 ± 19.7; and conductivity 424.6 ± 37.1 μS/cm. No significant differences in mortality were observed between groups during the exposure period and survival was > 88% in all groups.

### Confirmation of thyroid suppression: immunofluorescent labeling of thyroxine

At 7 dph, 10 larvae from each group larvae were euthanized via immersion in buffered MS-222 (0.3 g/L) and fixed in 4% paraformaldehyde overnight at 4 °C. Immunofluorescent labeling of T4 was performed according to Thienpont et al.^18^ with some modifications. Detailed methods can be found in the supplementary information.

### Confirmation of thyroid suppression: gene expression

At 30 dph, 8 larvae from each group were euthanized via immersion in buffered MS-222 (0.3 g/L). Liver tissue was removed, frozen on dry ice, and stored at − 80 °C. Methods for the extraction of total RNA, cDNA synthesis and qPCR can be found in the supplementary information. Here, transthyretin (*ttr*) and deiodinase 2 (*di2*) were targeted for gene expression analysis given their sensitivity to thyroid suppression^19^. Primer sequences and respective annealing temperatures are listed in Table S6. The expression of each target gene was normalized to acidic ribosomal protein (*arp*), which was found to be stable across groups.

### Confirmation of thyroid suppression: growth

Growth was assessed at 7 (n = 10/group) and 30 dph (n = 8/group) via the assessment of mass (wet weight) and length. Immediately following euthanasia, larvae were gently dried using a Kimwipe and weighed. Images of each larva were taken and total length (i.e., snout to tip of tail) was measured with ImageJ2 using the line tool^26^.

### Ex vivo immune cell assessment: respiratory burst and phagocytic cell activity

Respiratory burst and phagocytic cell activity were selected for the assessment of immune cell function given their importance to non-specific, innate immunity. Both assays were performed on cells harvested from the kidney according to methods outlined by Thornton Hampton et al.^27^. Respiratory burst was determined using a nitroblue tetrazolium reduction assay whereas phagocytic cell activity was assessed via the engulfment of fluorescein (FITC)-conjugated *Escherichia coli* K-12 BioParticles (Thermo Fisher Scientific, Waltham, MA) (n = 5/group). Detailed methods for the cell extraction and each assay can be found in the supplemental information.

### In vivo immune assessment: bacteria culture and injection procedure

At 5–7 months post hatch, adult minnows were injected with *Y. ruckeri* to assess the in vivo immune response and pathogen resistance. Methods for the pathogen resistance assay have been previously reported by Thornton et al.^16,28^. To create bacterial injection solutions, *Y. ruckeri* (ATCC 29473) were cultured in nutrient broth overnight (~ 14 h) on an orbital shaker at 26 °C. Bacteria were washed and resuspended in HBSS to generate a bacterial injection solution (OD_600_ = 0.8). Nutrient agar plating showed that the injection solution contained 1.35 × 10^9^ colony forming units (CFU)/mL. Immediately prior to injection, fish were anesthetized via immersion in buffered MS-222 (0.1 g/L) and weighed. Fish were administered 10 µL bacterial injection solution/g of body mass (1.35 × 10^7^ CFU/g body mass) via intraperitoneal injection using a syringe pump (NE-500, New Era Pump Systems, Inc., Farmingdale, NY) equipped with a 27-gauge needle. Following each injection, fish were immediately placed in 20 L aquaria and monitored to ensure recovery from anesthesia.

### In vivo immune assessment: tissue collection

The non-specific, innate immune response was assessed in a subset of fish (12/group) challenged with *Y. ruckeri*. Each fish was euthanized via immersion in buffered MS-222 (0.3 g/L) during an eight to ten-hour window following pathogen injection. Total body mass (wet weight) and length (i.e., snout to fork) were determined and peripheral blood was collected by severing the caudal artery and collecting blood in a heparinized microhematocrit tube. Kidney tissue was collected, placed on dry ice, and stored at − 80 °C for subsequent gene expression analysis. Spleen was weighed for the determination of spleen index ((tissue mass/total body mass) × 100).

### In vivo immune assessment: transcriptomic analysis

To assess differences in the renal transcriptomic response to *Y. ruckeri* infection following ELS thyroid suppression, four kidney samples were randomly selected from each treatment group. Detailed methods for RNA extraction, the preparation of cDNA libraries, sequencing, transcriptomic analysis, and qPCR validation can be found in the supplemental information. Transcriptome data may be accessed at the Gene Expression Omnibus website (GSE146105).

### In vivo immune assessment: leukocyte counts

Approximately 10 µL of blood was removed from each hematocrit tube using a micropipette to create a blood smear on a glass slide and air dried. Each slide was stained using Camco Quik Stain II (Cambridge Diagnostic Products, Fort Lauderdale, FL) according to manufacturer protocols. Representative images of each slide were taken using a Nikon Eclipse 90i microscope fitted with a DS-Fi1 camera and managed by NIS-Elements Advanced Research software v.4.6. Two hundred cells were counted using the cell counter plugin^29^ in ImageJ2^25^ by two independent researchers. Counts were then averaged together to determine the relative number of leukocytes to the total number of blood cells (n = 10–12/group).

### In vivo immune assessment: hematocrit

To assess hematocrit, blood which was not utilized for leukocyte counts was immediately centrifuged in microhematocrit tubes for ~ 2 min at room temperature according to methods by Bhamla et al.^30^. Photos of microhematocrit tubes were taken and hematocrit was determined by measuring the volume of packed erythrocytes relative to the total blood volume using the line tool in ImageJ2^26^ (n = 9–11/group).

### In vivo immune assessment: bacterial load

Bacterial load was assessed by measuring the relative abundance of the *Y. ruckeri*-specific 16S ribosomal subunit transcript in renal tissue following pathogen injection^15,31^. Total RNA isolation, cDNA synthesis and qPCR analysis were conducted as described for the validation of RNA-seq results. Methodological details may be found in the supplemental information. The primer sequences and annealing temperature are listed in Table S6.

### In vivo immune assessment: pathogen resistance

Fish were monitored for 14 d following pathogen injection for mortality and signs of infection (e.g., external hemorrhaging, lethargy, lack of appetite, etc.). Three independent pathogen resistance trials were conducted, each with 12 fish per treatment group.

### Statistical analyses

Phagocytic cell activity data were assessed via a two-way ANOVA with time and treatment group as factors followed by a Tukey’s post hoc multiple comparison test. To validate transcriptomics data, relative expression obtained via qPCR was compared to the CPM obtained via RNA sequencing analysis for each target gene using a regression analysis. For the results of the pathogen resistance assay, significant differences between groups were determined via a survival time analysis. For all other endpoints, significant differences between groups were determined via a one-way analysis of variance (ANOVA) followed by a Tukey’s post hoc multiple comparison test. In cases of unequal variance, significant differences were determined by a Kruskal–Wallis test followed by a Steel–Dwass post hoc multiple comparison test. Unless otherwise stated, all analyses were performed using the statistical software package JMP student edition 14.0. Statistical significance was set at α < 0.05 for analyses.

## Supplementary Information

Supplementary Information.

## References

[1] De Luca R (2021). Thyroid hormones interaction with immune response, inflammation and non-thyroidal illness syndrome. Front. Cell Dev. Biol..

[2] Jara EL (2017). Modulating the function of the immune system by thyroid hormones and thyrotropin. Immunol. Lett..

[3] Geven E, Klaren P (2017). The teleost head kidney: Integrating thyroid and immune signalling. Dev. Comp. Immunol..

[4] Quesada-García A (2014). Thyroid signaling in immune organs and cells of the teleost fish rainbow trout (*Oncorhynchus mykiss*). Fish. Shellfish Immunol..

[5] Quesada-García A (2016). Thyroid active agents T3 and PTU differentially affect immune gene transcripts in the head kidney of rainbow trout (*Oncorynchus mykiss*). Aquat. Toxicol..

[6] Rooney AA, Fournier M, Bernier J, Cyr DG (2003). Neonatal exposure to propylthiouracil induces a shift in lymphoid cell sub-populations in the developing postnatal male rat spleen and thymus. Cell. Immunol..

[7] Nakamura R (2007). Effects of developmental hypothyroidism induced by maternal administration of methimazole or propylthiouracil on the immune system of rats. Int. Immunopharmacol..

[8] Zhang Y (2017). Thyroid hormone regulates hematopoiesis via the TR-KLF9 axis. Blood.

[9] Lam SH, Sin YM, Gong Z, Lam TJ (2005). Effects of thyroid hormone on the development of immune system in zebrafish. Gen. Comp. Endocr..

[10] Nieto PA (2016). Gestational hypothyroidism improves the ability of the female offspring to clear *Streptococcus pneumoniae* infection and to recover from pneumococcal pneumonia. Endocrinology.

[11] Albornoz EA (2013). Gestational hypothyroidism increases the severity of experimental autoimmune encephalomyelitis in adult offspring. Thyroid.

[12] Haensgen H (2018). Gestational hypothyroxinemia affects its offspring with a reduced suppressive capacity impairing the outcome of the experimental autoimmune encephalomyelitis. Front. Immunol..

[13] Carr JA, Patiño R (2011). The hypothalamus–pituitary–thyroid axis in teleosts and amphibians: Endocrine disruption and its consequences to natural populations. Gen. Comp. Endocr..

[14] Fernández L, Méndez J, Guijarro JA (2007). Molecular virulence mechanisms of the fish pathogen *Yersinia ruckeri*. Vet. Microbiol..

[15] Raida M, Buchmann K (2008). Development of adaptive immunity in rainbow trout, *Oncorhynchus mykiss* (Walbaum) surviving an infection with *Yersinia ruckeri*. Fish. Shellfish. Immunol..

[16] Thornton LM (2017). Characterization of basic immune function parameters in the fathead minnow (*Pimephales promelas*), a common model in environmental toxicity testing. Fish. Shellfish Immunol..

[17] Cooper DS (2005). Antithyroid drugs. N. Engl. J. Med..

[18] Thienpont B (2011). Zebrafish eleutheroembryos provide a suitable vertebrate model for screening chemicals that impair thyroid hormone synthesis. Environ. Sci. Technol..

[19] Path, E. *Identifying Sensitive Endpoints of Thyroid Disruption in the Fathead Minnow After Exposure to Propylthiouracil*. Undergraduate Honors Thesis. Texas Christian University (2016).

[20] Sharma P, Grabowski TB, Patiño R (2016). Thyroid endocrine disruption and external body morphology of Zebrafish. Gen. Comp. Endocrinol..

[21] Reinwald H (2021). Toxicogenomic fin(ger)prints for thyroid disruption AOP refinement and biomarker identification in zebrafish embryos. Sci. Total Environ..

[22] Neumann NF, Stafford JL, Barreda D, Ainsworth AJ, Belosevic M (2001). Antimicrobial mechanisms of fish phagocytes and their role in host defense. Dev. Comp. Immunol..

[23] Esteban M, Cuesta A, Chaves-Pozo E, Meseguer J (2015). Phagocytosis in teleosts. Implications of the new cells involved. Biology.

[24] Geissmann F (2010). Development of monocytes, macrophages, and dendritic cells. Science.

[25] Sierra-Filardi E (2014). CCL2 Shapes macrophage polarization by GM-CSF and M-CSF: Identification of CCL2/CCR2-dependent gene expression profile. J. Immunol..

[26] Rueden CT (2017). Image J2: ImageJ for the next generation of scientific image data. BMC Bioinform..

[27] Thornton Hampton LM, Venables BJ, Sellin JM, K. (2020). A practical guide for assessing respiratory burst and phagocytic cell activity in the fathead minnow, an emerging model for immunotoxicity. MethodsX.

[28] Thornton LM, Path EM, Nystrom GS, Venables BJ, Sellin Jeffries MK (2018). Embryo-larval BDE-47 exposure causes decreased pathogen resistance in adult male fathead minnows (*Pimephales promelas*). Fish Shellfish Immunol..

[29] De Vos K. *Cell Counter ImageJ*. https://imagej.nih.gov/ij/plugins/cell-counter.html (2010).

[30] Bhamla MS (2017). Hand-powered ultralow-cost paper centrifuge. Nat. Biomed. Eng..

[31] Raida MK, Holten-Andersen L, Buchmann K (2011). Association between *Yersinia ruckeri* infection, cytokine expression and survival in rainbow trout (*Oncorhynchus mykiss*). Fish Shellfish Immunol..

