# Supplementary material for: Developmental thyroid disruption causes long-term impacts on immune cell function and transcriptional responses to pathogen in a small fish model
Source: Sci Rep. 2021 Jul 14;11:14496. doi: 10.1038/s41598-021-93929-8 (PMC8280131; doi:10.1038/s41598-021-93929-8)
Supplement: Supplementary file 1 — Supplementary Information. [file 41598_2021_93929_MOESM1_ESM.docx]

**Developmental thyroid disruption causes long-term impacts on immune cell function and transcriptional responses to pathogen in a small fish model**

Leah M. Thornton Hampton ^ab^, Miranda G. Finch ^a^, Christopher J. Martyniuk ^c^, Barney J. Venables ^b^ and Marlo K. Sellin Jeffries ^a^*

^a^Department of Biology, Texas Christian University, Fort Worth, Texas, USA

^b^Department of Biological Sciences, University of North Texas, Denton, Texas, USA

^c^Center for Environmental and Human Toxicology, Department of Physiological Sciences, College of Veterinary Medicine, UF Genetics Institute,

Interdisciplinary Program in Biomedical Sciences Neuroscience, University of Florida, Gainesville, Florida, USA

* Address correspondence to m.jeffries@tcu.edu; Department of Biology, Texas Christian University, 2800 S. University Dr. Fort Worth, TX 76129

Table S1. Mass and length of control and propylthiouracil (PTU)-exposed larvae at 7 (n=10) and 30 days post hatch (dph) (n=8) (mean ± standard error). Different letters indicate statistically significant differences between treatment groups.

|  | | **Control** | | | **Low PTU** | | | | **High PTU** | | | |  |
| --- | --- | --- | --- | --- | --- | --- | --- | --- | --- | --- | --- | --- | --- |
| **7dph** | |  |  |  | |  |  |  | |  |  |  | |
|  | Mass (mg) | 4.59 | ± | 0.21 | | 3.93 | ± | 0.18 | | 3.95 | ± | 0.23 | |
|  | Length (mm) | 9.19 | ± | 0.15 | | 8.84 | ± | 0.15 | | 9.09 | ± | 0.20 | |
|  |  |  |  |  | |  |  |  | |  |  |  | |
| **30dph** | |  |  |  | |  |  |  | |  |  |  | |
|  | Mass (mg) | 209.94 | ± | 0.03^a^ | | 165.29 | ± | 0.02^a^ | | 81.58 | ± | 0.02^b^ | |
|  | Length (mm) | 27.80 | ± | 0.47^a^ | | 25.41 | ± | 0.96^b^ | | 20.54 | ± | 0.34^c^ | |

Table S2. General morphology (mean ± standard error) of control and propylthiouracil (PTU)-exposed fish (n=5/group) utilized in the respiratory burst and phagocytic cell activity assays.

| **Endpoint** | **Control** | | | **Low PTU** | | | | **High PTU** | | | |  |
| --- | --- | --- | --- | --- | --- | --- | --- | --- | --- | --- | --- | --- |
| Mass (g)  Length (mm) | 2.18 | ± | 0.13 | | 2.49 | ± | 0.18 | | 2.10 | ± | 0.17 | |
|  | 52.61 | ± | 1.11 | | 55.75 | ± | 1.17 | | 52.73 | ± | 1.05 | |

Table S3. Respiratory burst, as measured by absorbance at 620 nm (mean ± standard error) in unstimulated and stimulated (phorbol 12-myristate 13-acetate) renal cell suspensions created from control and propylthiouracil (PTU)-exposed fish (n = 5/group).

|  | | **Control** | | | **Low PTU** | | | | **High PTU** | | | |  |
| --- | --- | --- | --- | --- | --- | --- | --- | --- | --- | --- | --- | --- | --- |
|  | Unstimulated | 0.07 | ± | 0.01 | | 0.04 | ± | 0.003 | | 0.09 | ± | 0.02 | |
|  | Stimulated | 0.24 | ± | 0.02 | | 0.22 | ± | 0.01 | | 0.23 | ± | 0.02 | |


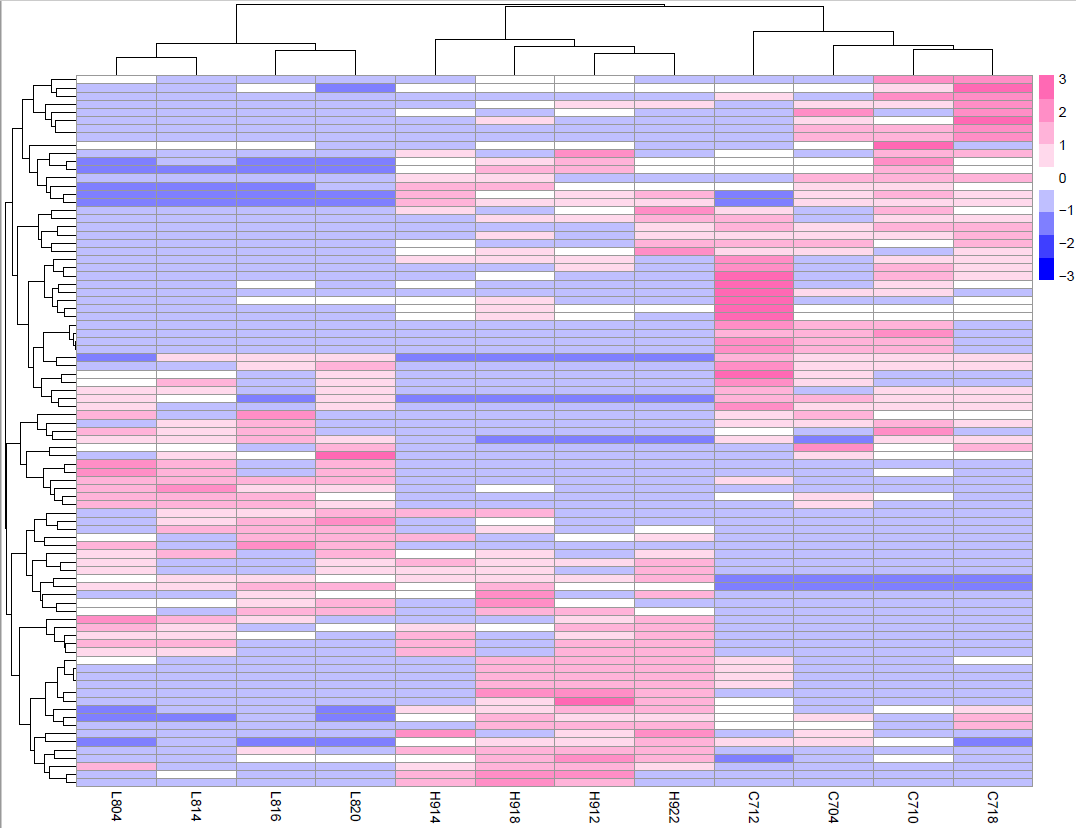


Figure S1. Clustering analysis of differentially expressed genes (counts per million) between individual samples from the control (C) low (L) and high (H) propylthiouracil (PTU) treatment groups. The color scale represents the z-score for each individual gene. Image produced using the pheatmap() package in R 3.6.1 (R Core team 2019).


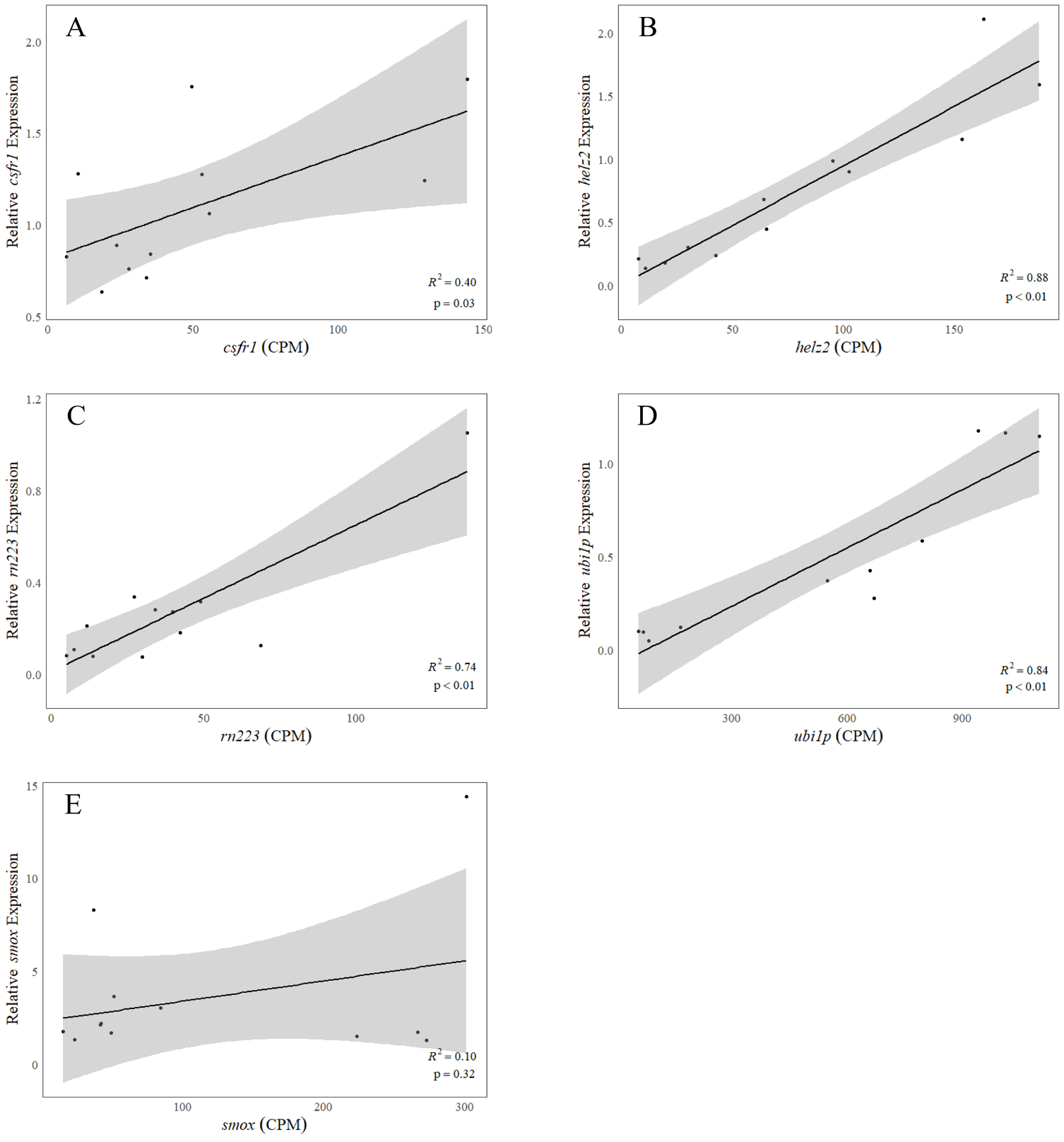


Figure S2. Validation of transcriptomic analysis via qPCR. Selected genes include (A) *csfr1* (colony stimulating factor receptor 1); (B) *helz2* (helicase with zinc finger domain 2; (C) *rn223* (RING finger protein 223); (D) *ubi1p* (polyubiquitin); (E) *smox* (spermine oxidase). The expression of each gene was normalized to the housekeeping gene actin-related protein and compared to the counts per million obtained via transcriptomics analysis.

Table S4. General morphology (mean ± standard error) of control and propylthiouracil (PTU)-exposed fish sampled at ~8-10 hours following pathogen injection (n = 11-12/group). Different letters indicate statistically significant differences between treatment groups.

| **Endpoint** | **Control** | | | **Low PTU** | | | | **High PTU** | | | |  |
| --- | --- | --- | --- | --- | --- | --- | --- | --- | --- | --- | --- | --- |
| Mass (g)  Length (mm) | 1.84 | ± | 0.19 | | 2.12 | ± | 0.18 | | 2.13 | ± | 0.18 | |
|  | 48.68 | ± | 1.54 | | 52.11 | ± | 1.36 | | 50.18 | ± | 1.31 | |

Table S5. Larval live *Artemia nauplii* feeding regime.

| **Larval Age (dph)** | **Artemia (mg, ww)/larva** |
| --- | --- |
| 2-6 | 3.19 |
| 7-8 | 4.79 |
| 9-14 | 7.18 |
| 15-17 | 10.77 |
| 18-19 | 16.15 |
| 20-30 | 24.22 |

*Abbreviations: days post hatch (dph), wet weight (ww)

*4.2 Confirmation of Thyroid Suppression - Immunofluorescent Labeling of Thyroxine*

At 7 dph, 10 larvae from each group larvae were euthanized via immersion in buffered MS-222 (0.3 g/L) and fixed in 4% paraformaldehyde overnight at 4°C. Immunofluorescent labeling of T4 was performed according to Thienpont et al. (2011) with some modifications. Specially, larvae were rinsed in blocking buffer for 3 h prior to incubation with the primary antibody, and each larva was incubated with the primary (1:500) and the secondary antibody (1:150) for ~96 h at 4°C. Larvae were imaged in glass bottom (No. 0 cover glass) petri dishes (MatTek Corporation, Ashland, MA) using a Zeiss Observer.Z1 equipped with a LSM 710 laser and ZEN 2009 imaging software. A Z stack was generated for each larva, which was then analyzed using ImageJ2 (Rueden et al. 2017). Specifically, the plugin StackReg (Thévenaz et al. 1998) was used to correct for horizontal drift that may have occurred during imaging and a maximum projection was generated from the stack. Integrated density (ID), which accounts for both fluorescence intensity as well as area, was measured according to Thienpont et al. 2011. Measurements of PTU-treated larvae normalized to the controls within each batch of samples that were processed together.

*4.3 RNA Extraction, cDNA Synthesis & qPCR*

At 30 dph, eight larvae from each group were euthanized via immersion in buffered MS-222 (0.3 g/L). Liver tissue was removed, frozen on dry ice and stored at -80°C. Tissues were homogenized using a QSonica tissue sonicator (QSonica, Farmingdale, NY), and total RNA was extracted from each sample using the Maxwell 16 LEV simplyRNA Purification Kit (Promega, Madison, WI) per manufacturer protocol as outlined in Sellin Jeffries et al. (2014). Total RNA was quantified and checked for purity using the NanoDrop 1000 (ThermoScientific, Wilmington, DE). All samples were of sufficient purity as indicated by each having 260/280 ratios ≥ 2.06. First-strand cDNA was synthesized using the iScript cDNA synthesis kit (BioRad, Hercules, CA). Each 10 µL reaction contained 2 µL of 5 x iScript Reaction mix, 0.5µL of iScript reverse transcriptase, and 0.1 µg of total RNA diluted into 7.5 µL of nuclease free water. Reactions were performed using a TC100 thermal cycler (BioRad, Hercules, CA) with a thermal cycling program of 5 min at 25°C, followed by 20min at 46°C, and 1 min at 95°C. All qPCR reactions were performed in triplicate using a CFX Connect real-time PCR detection system managed by CFX Manage Software, v.3.0 (BioRad, Hercules, CA). Each 10 µL qPCR reaction contained 5 µL SsoAdvanced Universal SYBR Green Supermix (BioRad, Hercules, CA), 0.3 µL of primer, 4.3 µL of nuclease free water and 0.4 µL of cDNA. The thermal cycling program consisted of an activation step (95°C, 3 min), followed by 40 cycles of denaturing (95°C, 10 s) and annealing (primer specific temperature, 30 s). A final melting curve was generated at the end of each cycling program to assess amplicon specificity. The expression of each gene was quantified via the standard curve method using serial diluted cDNA samples. Here, transthyretin (*ttr*) and deiodinase 2 (*dio2*) were targeted for gene expression analysis given their sensitivity to thyroid suppression (Path 2016). Primer sequences and respective annealing temperatures are listed in Table 4.2. The expression of each target gene was normalized to acidic ribosomal protein (*arp*), and statistical analysis revealed that there were no differences between treatment groups for the expression of *arp* throughout the present study (ANOVA, *p* value = 0.09).

Table S6. Primer sequences used in current study.

| **Gene^a^** | **Primer Sequences (5´→ 3´)** | **Annealing Temperature (°C)** | **Experimental Purpose** |
| --- | --- | --- | --- |
| *arp*  (AF134852.1) | FW: CTGAACATCTCGCCCTTCTC  RV: GACACACACTGGCGATGTTC | 60 | Reference Gene |
| *dio2*  (Thornton et al. 2018) | FW: AATTTTCGGATGTGGCAGAC  RV: GCAGCAAACATCCTCTCCTC | 61 | Confirmation of Thyroid suppression |
| *ttr*  (Chen et al. 2010) | FW: CTGGTGTGTATCGGGTGGAGTT  RV: GCATGAGCTTCAAACACCACAT | 61 | Confirmation of Thyroid suppression |
| *yr16s*  (Raida & Buchmann 2008) | FW: GCGAGGAGGAAGGGTTAAGTG  RV: GTTAGCCGGTGCTTCTTCTG | 60 | Bacterial Load |
| *csfr1* | FW: GATCATCAGCATCGGTCCTT  RV: TGGCAGTGAAAATGCTCAAG | 60 | Transcriptomic Validation |
| *helz2* | FW: TTTGCATCCATGCTCTTCAG  RV: CCAGATCATCCATTCCTGCT | 57 | Transcriptomic Validation |
| *rn223* | FW: TGTTGTATGCGGAAAAGCAG  RV: CTGAGCACACAACAGGAGGA | 60 | Transcriptomic Validation |
| *ubi1p* | FW: CTGGCTCTGTGGTGATGTTG  RV: GGACACTCTCTTTGCGGAAG | 60 | Transcriptomic Validation |
| *smox* | FW: AGCTTCTTGGCTGCAGAGAG  RV: GCAGGAAAACAGGAAGTGGA | 60 | Transcriptomic Validation |

^a^Abbreviations: *arp* (actin-related protein); *dio2* (deiodinase 2); *ttr* (transthyretin); *yr16s* (*Yersinia ruckeri* 16S ribosomal subunit); *csfr1* (colony stimulating factor receptor 1); *helz2* (helicase with zinc finger domain 2); *rn223* (RING finger protein 223); *ubi1p* (polyubiquitin); *smox* (spermine oxidase).

*4.5 Ex Vivo Immune Cell Assessment - Respiratory Burst and Phagocytic Cell Activity*

Respiratory burst and phagocytic cell activity were selected for the assessment of immune cell function given their importance to non-specific, innate immunity. Both assays were performed according to methods outlined by Thornton Hampton et al. 2020. Briefly, 15 fish per treatment group were euthanized via immersion in buffered MS-222 (0.3 g/L) at ~ 11 months post hatch. Mass and length were determined for each fish and each fish was exsanguinated by severing the caudal artery and collecting blood in a heparinized microhematocrit tube. Whole kidney tissue was dissected and immediately placed into 1.5 mL Eppendorf tubes (3 kidneys per tube) containing 100 µL supplemented Leibovitz’s L-15 cell media (5% fetal bovine serum, 1% penicillin/streptomycin, 1% 1.5 M HEPES and 0.5% L-Glutamine). Tissues were gently homogenized with a plastic pestle and filtered using a syringe loosely packed with glass wool. Cells were washed twice with supplemented L-15, counted using a hemocytometer and diluted to 6 x10^6^ cells/mL. Then, 100 µL of cell suspension was added to each well of a tissue culture treated 96-well flat bottom plate, and cells were allowed to recover overnight in a humidified incubator (30°C).

A nitroblue tetrazolium (NBT) reduction assay was utilized to assess respiratory burst with some modification (Secombes 1990, Choi et al. 2006). Briefly, respiratory burst was stimulated with 0.5 µg/mL PMA. The generation of reactive oxygen species by respiratory burst reduced NBT (0.8 mg/mL) to formazan. Following a one-hour incubation period at 30°C, wells were washed twice with 100 µL 70% MeOH and allowed to dry at room temperature. Formazan crystals were resolubilized with the addition of 120µL of 2M KOH followed by 140µL DMSO, and absorbance (620nm) was measured using a FLUOstar Omega Microplate Reader (BMG LABTECH, Cary, NC). All reactions were carried out in triplicate and mean values for blank wells (no cells) were subtracted from mean experimental well values.

To assess phagocytic cell activity, methods by Ninković and Roy (2014) were utilized with some modification. Briefly, fluorescein (FITC)-conjugated *Escherichia coli* K-12 BioParticles (Thermo Fisher Scientific, Waltham, MA) were added to cells in approximately a 10:1 ratio. Immediately afterwards, 50 µL of trypan blue (0.4%) was added to each well designated for the 0 h measurement and the plate was centrifuged at room temperature for 5 min at 500 rpm to synchronize phagocytosis by facilitating cell:particle contact. Fluorescence intensity (excitation filter: λ = 488 nm, emission filter: λ = 518 nm) was immediately measured using a FLUOstar Omega Microplate Reader (BMG LABTECH, Cary, NC). Cells were incubated at 30°C, and phagocytic cell activity being measured at 1, 2 and 4 hours by the addition of trypan blue followed by the measurement of fluorescence intensity as previously described. Each measurement was performed on three technical replicates and the value for mean fluorescence intensity at 0 hours was subtracted from all subsequent timepoints to account for background fluorescence.

*4.8 In Vivo Immune Assessment - Transcriptomic Analysis*

To assess differences in the renal transcriptomic response to *Y. ruckeri* infection following ELS thyroid suppression, four kidney samples were randomly selected from each treatment group. Total RNA was extracted, quantified and checked for purity as is previously described. RNA quality indicator (RQI) values were determined using the Experion RNA StdSens Kit in conjunction with the Experion Automated Electrophoresis System (BioRad, Hercules, CA) per manufacturer protocol. All samples had RQI values ≥ 9.0, indicating sufficient RNA quality for downstream analysis.

To prepare cDNA libraries, the NEBNext Ultra II RNA Library Prep kit in conjunction with the NEBNext poly(A) mRNA magnetic isolation module and NEBNext Multiplex Oligos for Illumina (New England BioLabs, Ipswich, MA) was utilized according to manufacturer protocols with an input of 500 ng total RNA. Library quality (e.g., fragment size, presence of adaptors) was assessed using the Experion DNA 1K Analysis kit in conjunction with the Experion Automated Electrophoresis System (BioRad, Hercules, CA). Finally, libraries were quantified using the NEBNext Library Quant Kit (BioRad, Hercules, CA) and shipped to University of Texas Health Science Center San Antonio where paired end sequencing (100 bp) was performed on the Illumina HiSeq 3000 platform.

Following sequencing, adaptor sequences and low-quality reads were removed from raw sequences using Trimmomatic v0.38 (Bolger et al. 2014). Specifically, trimming was applied using a sliding window approach with a minimum quality score of 20 followed by a minimum length filter of 36 bp as recommended by Williams et al. (2016) and Bolger et al. (2014). Libraries were assembled by combining two samples containing the greatest number of quality filtered reads from each of the following groups: sham-injected (HBSS, Thornton Hampton et al. *In Review*), control, low PTU and high PTU (a total of eight samples combined). A *de novo* assembly was generated with Trinity v2.4.0 using default settings (Haas et al. 2013). The *de novo* assembly was then annotated against the UniProtKB Swiss-Prot (www.uniprot.org) database using BLASTX with a cutoff E-value of less than 1.0 x 10^-5^. Each sample was then aligned to the *de novo* assembly using assembly using RNAseq by Expectation Maximization (RSEM v.1.3; Li & Dewey, 2011) to obtain gene-level counts. Significant differences between individual genes amongst all groups were determined using a Likelihood Ratio Test using edgeR (Robinson et al. 2010). Genes with an FDR-corrected p value < 0.05 or a log_2_FC < -1 or > 1 were determined to be significantly differentially expressed. Clustering analysis of DEGs using Pearson’s Correlation was performed using the Pretty Heatmap package v 1.0.12 in R (Kolde 2019).

Subnetwork enrichment analysis (SNEA) was conducted in Pathway Studio 11.0 (Elsevier) to determine gene networks enriched by ELS thyroid suppression following pathogen infection. To obtain fold-changes and p values to input into Pathway Studio, pairwise comparisons were made using a Fisher’s Exact Test in edgeR. Using the official gene name (Name + Alias), 70,045 genes were mapped to the program. Highest fold change, best p value was used for duplicated transcripts. The enrichment p value for gene networks was p < 0.05.

Finally, to validate results obtained by RNA-Seq analysis, the following transcripts were selected for targeted gene expression analysis via qPCR: *csfr1* (colony stimulating factor receptor 1); *helz2* (helicase with zinc finger domain 2); *rn223* (RING finger protein 223); *ubi1p* (polyubiquitin); *smox* (spermine oxidase). Additional genes were selected for targeted gene expression analysis, but the results were deemed unreliable due to low abundance (< ~40 CPM, data not shown). Total RNA isolation, cDNA synthesis and qPCR analysis were performed as previously described with the exception that first-strand cDNA was synthesized using qScript cDNA SuperMix (QuantaBio, Beverly, MA). Each 10 µL reaction contained 2 µL of qScript cDNA SuperMix and 0.1 µg of total RNA diluted into 7.5 µL of nuclease free water. Reactions were performed using a TC100 thermal cycler (BioRad, Hercules, CA) with a thermal cycling program of 5 min at 25°C, followed by 30 min at 42°C, and 5 min at 85°C. For qPCR reactions, PerfeCTa Sybr Green Fast Mix (QuantaBio, Beverly, MA) was utilized in place of SsoAdvanced Universal SYBR Green Supermix. Original primer sequences were designed using Primer 3 (http://biotools.umassmed.edu/bioapps/primer3_www.cgi). Optimal annealing temperature was determined for each original primer set by conducting qPCR reactions on a thermal gradient. Annealing temperature was selected based on greatest target amplicon yield. Primer sequences and respective annealing temperatures are listed in Table S6.

**Reference List**

Bolger, A. M.; Lohse, M.; Usadel, B. Trimmomatic: A Flexible Trimmer for Illumina Sequence Data. Bioinformatics 2014, 30 (15), 2114–2120. https://doi.org/10.1093/bioinformatics/btu170.

Choi, H.S.; Kim, J.W.; Cha Y.; Kim C. A quantitative nitroblue tetrazolium assay for determining intracellular superoxide anion production in phagocytic cells. Journal of Immunoassay & Immunochemistry 2006, 27(1), 31-44. https://doi.org/10.1080/15321810500403722.

Haas, B. J.; Papanicolaou, A.; Yassour, M.; Grabherr, M.; Blood, P. D.; Bowden, J.; Couger, M. B.; Eccles, D.; Li, B.; Lieber, M.; et al. De Novo Transcript Sequence Reconstruction from RNA-Seq Using the Trinity Platform for Reference Generation and Analysis. Nature Protocols 2013, 8 (8), 1494–1512. https://doi.org/10.1038/nprot.2013.084.

Kolde, R. pheatmap v.1.0.12 2019. https://cran.r-project.org/package=pheatmap.

Li, B.; Dewey, C. N. RSEM: Accurate Transcript Quantification from RNA-Seq Data with or without a Reference Genome. BMC Bioinformatics 2011, 16.

Nieto, P. A.; Peñaloza, H. F.; Salazar-Echegarai, F. J.; Castellanos, R. M.; Opazo, M. C.; Venegas, L.; Padilla, O.; Kalergis, A. M.; Riedel, C. A.; Bueno, S. M. Gestational Hypothyroidism Improves the Ability of the Female Offspring to Clear *Streptococcus Pneumoniae* Infection and to Recover From Pneumococcal Pneumonia. Endocrinology 2016, 157 (6), 2217–2228. https://doi.org/10.1210/en.2015-1957.

Ninković, J.; Roy, S. High Throughput Fluorometric Technique for Assessment of Macrophage Phagocytosis and Actin Polymerization. Journal of Visual Experimentation 2014, (93), e52195. https://doi.org/10.3791/52195.

Path, E. Identifying sensitive endpoints of thyroid disruption in the fathead minnow after exposure to propylthiouracil. Undergraduate Honors Thesis. Texas Christian University, Fort Worth, Texas 2016.

Quesada-García, A.; Valdehita, A.; Kropf, C.; Casanova-Nakayama, A.; Segner, H.; Navas, J. M. Thyroid Signaling in Immune Organs and Cells of the Teleost Fish Rainbow Trout (*Oncorhynchus mykiss*). Fish & Shellfish Immunology 2014, 38 (1), 166–174. https://doi.org/10.1016/j.fsi.2014.03.016.

Quesada-García, A.; Encinas, P.; Valdehita, A.; Baumann, L.; Segner, H.; Coll, J. M.; Navas, J. M. Thyroid Active Agents T3 and PTU Differentially Affect Immune Gene Transcripts in the Head Kidney of Rainbow Trout (*Oncorynchus mykiss*). Aquatic Toxicology 2016, 174, 159–168. https://doi.org/10.1016/j.aquatox.2016.02.016.

R Core Team. R: A language and environment for statistical computing. R Foundation for Statistical Computing, Vienna, Austria, 2019. https://www.R-project.org/.

Raida, M.; Buchmann, K. Development of Adaptive Immunity in Rainbow Trout, *Oncorhynchus mykiss* (Walbaum) Surviving an Infection with *Yersinia ruckeri*. Fish & Shellfish Immunology 2008, 25 (5), 533–541. https://doi.org/10.1016/j.fsi.2008.07.008.

Raida, M. K.; Holten-Andersen, L.; Buchmann, K. Association between *Yersinia ruckeri* Infection, Cytokine Expression and Survival in Rainbow Trout (*Oncorhynchus mykiss*). Fish & Shellfish Immunology 2011, 30 (6), 1257–1264. https://doi.org/10.1016/j.fsi.2011.03.022.

Robinson, M. D.; McCarthy, D. J.; Smyth, G. K. EdgeR: A Bioconductor Package for Differential Expression Analysis of Digital Gene Expression Data. Bioinformatics 2010, 26 (1), 139–140. https://doi.org/10.1093/bioinformatics/btp616.

Rooney, A. A.; Fournier, M.; Bernier, J.; Cyr, D. G. Neonatal Exposure to Propylthiouracil Induces a Shift in Lymphoid Cell Sub-Populations in the Developing Postnatal Male Rat Spleen and Thymus. Cellular Immunology 2003, 223 (2), 91–102. https://doi.org/10.1016/S0008-8749(03)00153-9.

Rueden, C. T.; Schindelin, J. Hiner, M. C. DeZonia B.E.; Walter A.E.; Arena E.T.; Eliceiri K.W. ImageJ2: ImageJ for the next generation of scientific image data. BMC Bioinformatics 2017, 18 (529) https://doi:10.1186/s12859-017-1934-z.

Secombes, C.J. Isolation of salmonid macrophages and analysis of their killing activity. In: Techniques in Fish Immunology 1990, 137-155.

Sharma, P.; Grabowski, T. B.; Patiño, R. Thyroid Endocrine Disruption and External Body Morphology of Zebrafish. General and Comparative Endocrinology 2016, 226, 42–49. https://doi.org/10.1016/j.ygcen.2015.12.023.

Sierra-Filardi, E.; Nieto, C.; Domínguez-Soto, Á.; Barroso, R.; Sánchez-Mateos, P.; Puig-Kroger, A.; López-Bravo, M.; Joven, J.; Ardavín, C.; Rodríguez-Fernández, J. L.; et al. CCL2 Shapes Macrophage Polarization by GM-CSF and M-CSF: Identification of CCL2/CCR2-Dependent Gene Expression Profile. The Journal of Immunology 2014, 192 (8), 3858–3867. https://doi.org/10.4049/jimmunol.1302821.

Thévenaz, P.; Ruttimann, U.E.; Unser, M. A Pyramid Approach to Subpixel Registration Based on Intensity. IEEE Transactions on Image Processing 1998, 7 (1), 27-41.

Thienpont, B.; Tingaud-Sequeira, A.; Prats, E.; Barata, C.; Babin, P. J.; Raldúa, D. Zebrafish Eleutheroembryos Provide a Suitable Vertebrate Model for Screening Chemicals That Impair Thyroid Hormone Synthesis. Environmental Science & Technology 2011, 45 (17), 7525–7532. https://doi.org/10.1021/es202248h.

Thornton, L. M.; LeSueur, M. C.; Yost, A. T.; Stephens, D. A.; Oris, J. T.; Sellin Jeffries, M. K. Characterization of Basic Immune Function Parameters in the Fathead Minnow (*Pimephales promelas*), a Common Model in Environmental Toxicity Testing. Fish & Shellfish Immunology 2017, 61, 163–172. https://doi.org/10.1016/j.fsi.2016.12.033.

Thornton, L. M.; Path, E. M.; Nystrom, G. S.; Venables, B. J.; Sellin Jeffries, M. K. Embryo-Larval BDE-47 Exposure Causes Decreased Pathogen Resistance in Adult Male Fathead Minnows (*Pimephales promelas*). Fish & Shellfish Immunology 2018, 80, 80–87. https://doi.org/10.1016/j.fsi.2018.05.059.

Thornton Hampton, L. M., Venables, B. J., Sellin Jeffries M. K. A practical guide for assessing respiratory burst and phagocytic cell activity in the fathead minnow, an emerging model for immunotoxicity. MethodsX, 2020, In Press. https://doi.org/10.1016/j.mex.2020.100992.

Williams, C. R.; Baccarella, A.; Parrish, J. Z.; Kim, C. C. Trimming of Sequence Reads Alters RNA-Seq Gene Expression Estimates. BMC Bioinformatics 2016, 17 (1), 103. https://doi.org/10.1186/s12859-016-0956-2.

Zhang, Y.; Xue, Y.; Cao, C.; Huang, J.; Hong, Q.; Hai, T.; Jia, Q.; Wang, X.; Qin, G.; Yao, J.; et al. Thyroid Hormone Regulates Hematopoiesis via the TR-KLF9 Axis. Blood 2017, 130 (20), 2161–2170. https://doi.org/10.1182/blood-2017-05-783043.
